# Reliable Genotypic Tropism Tests for the Major HIV-1 Subtypes

**DOI:** 10.1038/srep08543

**Published:** 2015-02-25

**Authors:** Kieran Cashin, Lachlan R. Gray, Katherine L. Harvey, Danielle Perez-Bercoff, Guinevere Q. Lee, Jasminka Sterjovski, Michael Roche, James F. Demarest, Fraser Drummond, P. Richard Harrigan, Melissa J. Churchill, Paul R. Gorry

**Affiliations:** 1 Center for Biomedical Research, Burnet Institute, Melbourne, Australia 3004; 2 Department of Microbiology and Immunology, University of Melbourne, Parkville, Australia 3010; 3 Department of Infectious Diseases, Monash University, Melbourne, Australia 3800; 4 Laboratory of Retrovirology, Centre Recherche Public de la Santé, Luxembourg 1526; 5 BC Centre for Excellence in HIV/AIDS, Vancouver, Canada Y6Z 1Y6; 6 ViiV Healthcare, Research Triangle Park, North Carolina, USA 27709-3398; 7 ViiV Healthcare Australia, Abbotsford, Australia 3067; 8 Department of Medicine, Monash University, Melbourne, Australia 3800; 9 Department of Microbiology, Monash University, Melbourne, Australia 3800; 10 School of Applied Sciences, College of Science, Engineering and Health, RMIT University, Melbourne, Australia 3001

## Abstract

Over the past decade antiretroviral drugs have dramatically improved the prognosis for HIV-1 infected individuals, yet achieving better access to vulnerable populations remains a challenge. The principal obstacle to the CCR5-antagonist, maraviroc, from being more widely used in anti-HIV-1 therapy regimens is that the pre-treatment genotypic “tropism tests” to determine virus susceptibility to maraviroc have been developed primarily for HIV-1 subtype B strains, which account for only 10% of infections worldwide. We therefore developed PhenoSeq, a suite of HIV-1 genotypic tropism assays that are highly sensitive and specific for establishing the tropism of HIV-1 subtypes A, B, C, D and circulating recombinant forms of subtypes AE and AG, which together account for 95% of HIV-1 infections worldwide. The PhenoSeq platform will inform the appropriate use of maraviroc and future CCR5 blocking drugs in regions of the world where non-B HIV-1 predominates, which are burdened the most by the HIV-1 pandemic.

Since 2005, AIDS related deaths have fallen by 30%, largely due to the rollout of highly effective antiretroviral therapies. However, achieving adequate global access to antiretrovirals remains a major challenge, with approximately 66% of HIV-1 infected individuals considered eligible for therapy unable to access treatment^1^. One barrier to drug accessibility is the need for specialized prognostic laboratory tests to inform the appropriate use of anti-HIV-1 drugs.

Maraviroc is the first licensed drug in a relatively new class of HIV-1 entry inhibitors called CCR5 antagonists^2^. Entry of HIV-1 into cells of the immune system is initiated by primary engagement of the CD4 receptor on the cell surface, then secondary engagement of either of the chemokine coreceptors, CCR5 or CXCR4. Maraviroc blocks the ability of HIV-1 to engage CCR5, which inhibits viral entry into cells^2^, but does not block engagement of CXCR4 which is used by a substantial proportion of circulating virus, particularly at later stages of infection^3^,^4^,^5^,^6^. A specialized pre-treatment prognostic (or “tropism”) test is therefore mandatory to exclude patients with detectable CXCR4-using virus from being treated with CCR5 antagonists^7^,^8^,^9^.

Traditional “phenotypic” tropism tests for establishing HIV-1 coreceptor specificity use recombinant viruses pseudotyped with patient derived HIV-1 envelope proteins to infect cell lines expressing CD4 and either CCR5 or CXCR4^9^,^10^,^11^,^12^,^13^. However, on a global scale the cost, lengthy turn-around time, and highly specialized nature of these tests have been obstacles to maraviroc being used more widely in HIV-1 treatment regimens.

In contrast, “genotypic” coreceptor usage prediction algorithms, trained on characteristic HIV-1 sequence alterations within the third variable region (V3) of the viral envelope gene (*env*) offer a comparatively inexpensive, rapid and accessible alternative to phenotypic tropism assays. However, while genotypic algorithms can be highly sensitive for predicting coreceptor usage and treatment outcome for patients receiving maraviroc^9^,^10^,^11^,^13^,^14^,^15^,^16^,^17^,^18^,^19^,^20^, the majority were trained on HIV-1 subtype B V3 sequences, and consequently they have limited capacity for establishing coreceptor usage of non-B HIV-1 subtypes that have distinctive V3 sequences, and which account for approximately 90% of infections worldwide^13^,^14^,^21^,^22^,^23^. This has particular implications for regions of the world where non-B HIV-1 strains predominate, and which have expanding economies and rapidly improving health care systems such as Eastern Europe and Russia where HIV-1 subtype A is endemic, India and China where HIV-1 subtype C predominates^24^, and Thailand, Indonesia and Vietnam where a circulating recombinant form of HIV-1 subtypes A and E predominates (referred to as HIV-1 CRF01_AE)^25^,^26^. Furthermore, HIV-1 CRF01_AE is becoming more prevalent in developed countries such as Japan and Singapore^27^,^28^. These are all populous regions with moderate to high HIV-1 burdens. These factors constitute an economic and clinical environment suitable for the introduction of CCR5 antagonists to HIV-1 treatment regimens, with significant potential to benefit large HIV-1 affected populations. The lack of genotypic algorithms designed specifically for non-B HIV-1 subtypes is presently a major barrier to informing the appropriate use of maraviroc for treatment of HIV-1 infected individuals from these regions, and will continue to be a barrier for new coreceptor blocking drugs as they are developed.

Here, we report the development and utility of PhenoSeq, a suite of new genotypic algorithms that are highly sensitive and specific for establishing coreceptor usage of HIV-1 subtypes A, B, C, D, CRF01_AE and of a circulating recombinant form of HIV-1 subtypes A and G (referred to as HIV-1 CRF02_AG), which together account for approximately 95% of HIV-1 strains worldwide (15% subtype A, 10% subtype B, 50% subtype C, 5% subtype D, 5% CRF01_AE and 10% CRF02_AG). Our free-to-use, automated online platform of prognostic tools (www.burnet.edu.au/phenoseq) will inform the appropriate use of maraviroc and future coreceptor blocking drugs in regions of the world where non-B HIV-1 strains predominate.

## Results

### Developing PhenoSeq algorithms

**Figure 1:**
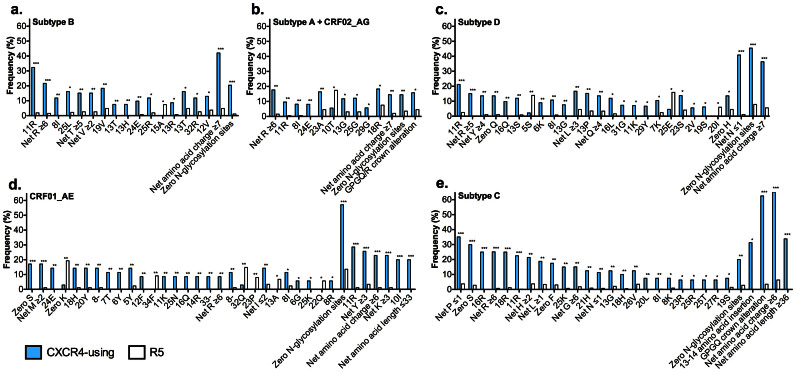
Training set V3 characteristics and amino acid mutations associated with coreceptor usage. Sequence analysis results demonstrating amino acid alterations that differentiate phenotypically characterized CXCR4-using from R5 V3 sequences are shown for (a) HIV-1 subtype B (n = 93 CXCR4-using and n = 296 R5 sequences), (b) HIV-1 subtype A and CRF02_AG (n = 60 CXCR4-using and n = 172 R5 sequences), (c) HIV-1 CRF01_AE (n = 50 CXCR4-using and n = 128 R5 sequences), (d) HIV-1 subtype D (n = 57 CXCR4-using and n = 80 R5 sequences) and (e) HIV-1 subtype C (n = 80 CXCR4-using and n = 429 R5 sequences); “GPGQ/R crown alteration” refers to V3 sequences where residues 16–19 are not GPGQ or GPGR; “GPGQ crown alteration” refers to V3 sequences where residues 16–19 are not GPGQ; “13–14 amino acid insertion” refers to V3 sequences with a two amino acid insertion between residues 13 and 14. P-values for net amino acid length, net amino acid charge and number of glycosylation sites were calculated using a Mann Whitney U-test (two-tailed). P-values for amino acid alterations were calculated using a Fisher's exact t-test (two-tailed). *** p-value < 0.0001, ** p < 0.01, * p < 0.05. Notably, a number of these V3 amino acid alterations have been associated with HIV-1 coreceptor usage^3^,^21^,^23^,^29^,^32^,^38^,^40^,^41^,^58^,^60^,^61^,^62^,^63^,^64^,^65^,^66^,^67^,^68^,^69^,^70^,^71^,^72^,^73^,^74^,^75^,^76^.

To develop the PhenoSeq algorithms, we first analyzed “training sets” comprising all available HIV-1 V3 sequences from the Los Alamos HIV-1 Database that had corresponding subtype and phenotypic tropism test results, to elucidate statistically significant alterations that distinguish CXCR4-using from CCR5-using (R5) viruses. Notably, we selected one sequence per phenotype per patient in order to avoid bias by resampling similar V3 sequences from a single HIV-1 infected individual. We focused on the V3 region since it is the major HIV-1 sequence determinant of coreceptor specificity^29^,^30^. For these analyses, we evaluated HIV-1 V3 amino acid length, net amino acid charge, number of N-linked glycosylation sites and the frequency of site-specific amino acid alterations. These sequence analysis results are summarized in Figure 1. Notably, because CRF02_AG strains contain a subtype A-like V3 region, we pooled and analyzed CRF02_AG and subtype A sequences together (Fig. 1b), and developed a single subtype A and CRF02_AG specific PhenoSeq algorithm (PhenoSeq-A/AG).

A unique feature of the PhenoSeq design method is the provision for continuous improvement and refinement of prediction criteria as new V3 sequences become available, to maintain maximal predictive accuracy. To illustrate this, here we re-evaluated and optimized our recently described HIV-1 subtype C specific algorithm CoRSeq_V3-C_^21^ (now re-named PhenoSeq-C), by adding 27 CXCR4-using and 35 R5 V3 sequences to the original CoRSeq_V3-C_ training set, which have been made available since its development (Fig. 1e).

The coreceptor usage prediction criteria for each PhenoSeq algorithm was determined by testing all combinations of V3 sequence alterations and selecting the combinations with the fewest V3 sequence alterations that, when tested against their respective training set sequences, optimized their sensitivity for detecting CXCR4-using HIV-1, without compromising specificity for detecting R5 strains (Table 1). Notably, we selected the most accurate combination of prediction parameters that comprised the fewest V3 sequence alterations in order to minimize the potential for including parameters that were unique to the PhenoSeq training sets. For simplicity, only comparisons to the clinically validated Geno2Pheno (g2p) [at false positive rates (FPR) of 5.75% and 10%], WebPSSM_X4R5_ and WebPSSM_SI/NSI_ algorithms are shown^15^,^31^,^32^. Extended comparisons to all the alternative algorithms and prediction rules that have been described in the literature are shown in Supplementary Tables 1–5. To demonstrate the optimization of the PhenoSeq prediction criteria, Table 1 shows that PhenoSeq-B, PhenoSeq-C, PhenoSeq-D, PhenoSeq-AE and PhenoSeq-A/AG are the most sensitive algorithms for detecting CXCR4-usage of the HIV-1 training set sequences without compromising specificity. The PhenoSeq-B, PhenoSeq-D, PhenoSeq-C, PhenoSeq-AE and PhenoSeq-A/AG prediction criteria are illustrated in Supplementary Figures 1–5.

### Developing the bioinformatics tool bulk2clonal

Validating the predictive accuracy of the PhenoSeq algorithms in a clinically relevant setting requires testing their performance against sets of phenotypically characterized V3 sequences derived from plasma of individuals infected with HIV-1 subtypes A, B, C, D, CRF01_AE or CRF02_AG that are independent of the training sets of Los Alamos HIV Database derived V3 sequences. However, to do this we first needed to develop a new program that would make the PhenoSeq algorithms compatible with V3 sequences generated by routine diagnostic laboratories.

This is because, when selecting our PhenoSeq training set sequences from the Los Alamos HIV Database, it was necessary to exclude V3 sequences that contained sites of base-call ambiguity, since this would impede analyses of V3 characteristics. Consequently, at this point of development, the predictive accuracy of PhenoSeq algorithms is dependent on unambiguous query V3 sequences. Unambiguous V3 sequences can be isolated from patient blood samples using contemporary clonal and next-generation deep sequencing techniques, however in the clinical setting, the majority of V3 sequences are isolated by much less expensive Sanger (or “bulk”) sequencing techniques. Bulk V3 sequencing produces a consensus sequence whereby each nucleotide represents the most frequent base at a given position within the sampled HIV-1 quasispecies. Consequently, bulk sequencing often produces sequences with sites of ambiguity whereby two or more nucleotides occur with equal frequency. Therefore, to enable compatibility with bulk V3 sequencing techniques we interfaced each of the PhenoSeq algorithms with a new bioinformatic tool that we developed, called “bulk2clonal”.

Briefly, bulk2clonal converts nucleotide V3 sequences containing ambiguity into multiple, unambiguous amino acid sequences, by generating and translating all possible nucleotide combinations, as described in the Methods (Supplementary Fig. 6). We configured PhenoSeq algorithms to predict a bulk V3 sequence containing ambiguity to be CXCR4-using if ≥10% of the protein sequences generated by bulk2clonal were predicted to be CXCR4-using. PhenoSeq then predicts a patient to harbour CXCR4-using HIV-1 if one or more bulk V3 nucleotide sequences isolated from that patient are predicted to be CXCR4-using.

### Validating PhenoSeq algorithms integrated with bulk2clonal

To validate the predictive accuracy of each PhenoSeq algorithm (now integrated with bulk2clonal) in a clinically relevant setting, we next compared their sensitivity, specificity and area under the receiver operating characteristic curve (AUROC) to several alternate algorithms for predicting the coreceptor usage of panels of bulk V3 sequences isolated from patient plasma samples that are unavailable on the Los Alamos HIV Database and thus independent of PhenoSeq training sets, relative to known phenotypic tropism assay results. Statistical comparison of AUROC scores was performed to assess whether the PhenoSeq algorithms were statistically more accurate than alternative genotypic algorithms, as described by Hanley *et al*^33^.

PhenoSeq-B was tested against 12 CXCR4-using and 41 R5 bulk V3 sequences from patients of a study by Mulinge *et al*^13^ that were phenotyped by an in-house recombinant virus phenotypic tropism assay (RVA) (Table 2; PhenoSeq-B Test Set 1). PhenoSeq-B demonstrated the highest possible sensitivity (100%), without compromising specificity (87.8%), and an AUROC that was statistically greater than WebPSSM_X4R5_ (p < 0.01) and WebPSSM_SI/NSI_ (p < 0.01), and statistically similar to g2p at FPRs of 5.75% and 10%. Considering the relatively low number of CXCR4-using V3 sequences used here, we also tested PhenoSeq-B against 92 CXCR4-using and 269 R5 independent V3 sequences from the Los Alamos HIV Database (Table 2; Test Set 2). Here, PhenoSeq demonstrated the highest sensitivity (78.4%), without compromising specificity (80.3%), and an AUROC that was statistically similar to g2p at FPRs of 5.75% and 10%, and WebPSSM_X4R5_ and WebPSSM_SI/NSI_.

We next tested the newly-optimized PhenoSeq-C algorithm against 55 CXCR4-using and 40 R5 bulk V3 sequences from 95 participants of the Pfizer epidemiology trial A4001064 that were phenotyped by the original Trofile™ phenotypic tropism assay (OTA)^14^,^34^, and against 18 CXCR4-using and 187 R5 bulk V3 sequences from 205 participants of the Pfizer phase III Maraviroc versus Efavirenz in Treatment-Naïve Patients (MERIT) trial that were phenotyped by the enhanced sensitivity Trofile™ phenotypic tropism assay (ESTA)^35^ (Table 2; PhenoSeq-C Test Sets 1 and 2, respectively). For the A4001064 patients, PhenoSeq-C demonstrated the highest sensitivity (83.6%), without compromising specificity (92.5%), and an AUROC that was statistically similar to g2p at FPRs of 5.75% and 10%, and WebPSSM_SI/NSI_-C. For the MERIT patients, PhenoSeq-C demonstrated the highest sensitivity (77.8%), without compromising specificity (75.4%), and an AUROC that was statistically greater than g2p at a FPR of 5.75% (p < 0.01), and which was similar to g2p at a FPR of 10% and WebPSSM_SI/NSI_-C.

PhenoSeq-D was tested against 43 CXCR4-using and 44 R5 bulk V3 sequences from 87 A4001064 participants that were phenotyped by OTA^14^,^34^ (Table 2; PhenoSeq-D Test Set). Our results show that PhenoSeq-D had the most favorable sensitivity (80.5%) and specificity (77.3%) profile and the highest AUROC, which was statistically similar to g2p at FPRs of 5.75% and 10%, WebPSSM_X4R5_ and WebPSSM_SI/NSI_.

PhenoSeq-AE was tested against 14 CXCR4-using and 25 R5 bulk V3 sequences from 39 patients phenotyped by RVA^13^ (Table 2; PhenoSeq-AE Test Set). Our results show that PhenoSeq-AE had the highest sensitivity (85.7%) without compromising specificity (96%), and an AUROC that was statistically greater than g2p at FPRs of 5.75% (p = 0.046) and 10% (p = 0.03).

Finally, PhenoSeq-A/AG was tested against 9 CXCR4-using and 69 R5 bulk V3 sequences from 78 A4001064 participants that were phenotyped by OTA^14^,^34^ (Table 1; PhenoSeq-A/AG Test Set 1), and 8 CXCR4-using and 36 R5 bulk V3 sequences from 44 patients that were phenotyped by RVA^13^ (Table 2; PhenoSeq-A/AG Test Set 2). For both test sequence sets, PhenoSeq-A/AG demonstrated the most favorable sensitivity (88.9% and 62.5%, respectively) and specificity (76.8% and 97.2%, respectively) profiles, and had the highest AUROC.

## Discussion

Maraviroc is the first licensed drug in a relatively new class of anti-HIV-1 therapeutics called CCR5 antagonists, which bind to CCR5 and block CCR5-mediated HIV-1 entry into cells. Since maraviroc is ineffective against CXCR4-using HIV-1, a coreceptor usage (or “tropism”) test is required before its administration. The most frequently used phenotypic tropism test is the enhanced sensitivity Trofile™ assay (ESTA)^35^,^36^,^37^, yet several factors such as cost and turn-around time have limited the widespread clinical use of ESTA and other phenotypic tropism assays, which in turn has limited the access of maraviroc to many eligible patients. On the other hand, genotypic algorithms enable most diagnostic laboratories to establish HIV-1 coreceptor usage by amplifying and sequencing the relatively short V3 region of *env* from patient blood samples, which compared to phenotypic tropism assays is a relatively inexpensive, rapid and straightforward process. Unfortunately, the majority of the currently available genotypic algorithms have been developed against HIV-1 subtype B V3 sequences and consequently they lack optimal predictive accuracy against non-B HIV-1 V3 sequences, as many of the V3 loop determinants of coreceptor specificity are subtype specific^3^,^13^,^22^,^23^,^29^,^38^,^39^,^40^,^41^,^42^,^43^,^44^,^45^,^46^,^47^. The lack of reliable genotypic algorithms that have been designed specifically for non-B HIV-1 subtypes is presently a major barrier to informing the appropriate use of maraviroc and future HIV-1 coreceptor blocking drugs in subjects infected with non-B HIV-1, which comprise approximately 90% of infections worldwide.

Here, we have conducted the most extensive and comprehensive analysis of phenotypically characterized HIV-1 subtype A, B, C, D, CRF01_AE and CRF02_AG V3 sequences to date, and developed subtype specific genotypic algorithms that are highly sensitive for predicting CXCR4-usage of HIV-1 in a clinical setting, without compromising specificity. Furthermore, we report the development and utility of a novel bioinformatic tool termed bulk2clonal, which computes and translates every possible amino acid sequence from nucleotide V3 sequences containing sites of base-call ambiguity. We showed that each of the PhenoSeq algorithms, when interfaced with bulk2clonal, are highly sensitive and specific for predicting CXCR4-usage of clinically relevant independent plasma-derived bulk V3 sequences that were generated by routine diagnostic laboratories. The performance of PhenoSeq-C against the MERIT clinical trial samples was particularly revealing. Of the 205 C-HIV infected individuals previously enrolled in MERIT, 18 belonged to a unique subset that was initially determined to harbor only R5 viruses by OTA, but then after failing maraviroc therapy were retrospectively shown to have harbored low frequency CXCR4-using strains by ESTA^10^,^35^,^48^,^49^. PhenoSeq-C detected minor CXCR4-using variants in 14 of these 18 subjects (accuracy 77.8%), thus correctly predicting their maraviroc treatment failure. These findings further demonstrate that our novel approach to genotypic tropism testing is highly sensitive and clinically valuable.

For determining coreceptor usage of HIV-1 subtype A and CRF02_AG, although PhenoSeq-A/AG exhibited a more favorable sensitivity and specificity profile than the clinically validated g2p at FPRs of 5.75% and 10%, WebPSSM_X4R5_ and WebPSSM_SI/NSI_, the recently developed HIVcoPRED (SAAC) and HIVcoPRED (SAAC + BLAST) algorithms exhibited the most favorable sensitivity (88% and 90%, respectively) and specificity (both 85.2%) profiles when tested against the PhenoSeq-A/AG training set sequences that were obtained from the Los Alamos HIV database (Supplementary Table 4). However, the performance of both of the HIVcoPRED algorithms was relatively poor compared to PhenoSeq-A/AG when tested against clinically relevant patient-derived bulk V3 sequences, even when they were coupled with bulk2clonal (Supplementary Table 4). These findings may be explained by the fact that HIVcoPRED (SAAC) and HIVcoPRED (SAAC + BLAST) training sets consisted of HIV-1 subtype A and CRF02_AG V3 sequences sampled from the Los Alamos HIV Database^50^, many of which were likely used here to test these algorithms.

Given that the PhenoSeq platform is highly sensitive and specific for predicting CXCR4-using HIV-1 strains, it is likely to be clinically useful for predicting treatment outcome for patients receiving maraviroc or indeed future CCR5 antagonists as they are developed. However, we acknowledge that the sensitivity and specificity of PhenoSeq for correctly determining HIV-1 coreceptor usage was measured against the results of phenotypic tropism assays rather than against maraviroc treatment outcome. In future studies we plan to determine the ability of the PhenoSeq platform to retrospectively predict virological outcome in patients who received maraviroc in the Pfizer phase III clinical trials MOTIVATE, MERIT and A4001029 using plasma-derived V3 sequences isolated by bulk and deep sequencing techniques^7^,^35^,^49^,^51^,^52^,^53^. For this study, we arbitrarily assigned a liberal cut-off of ≥10% for the analysis of amino acid sequences generated by bulk2clonal in order to maintain high sensitivity for predicting CXCR4-usage without compromising specificity. Through these planned studies we will more precisely determine the clinically relevant bulk2clonal cut-off required to accurately predict virological outcome in patients receiving maraviroc.

PhenoSeq is the first open access, online suite of genotypic algorithms to offer coreceptor usage analyses specifically designed for the major HIV-1 subtypes, which together account for approximately 95% of circulating viruses worldwide. Furthermore, the provision for constant revision and optimization of the PhenoSeq predictive criteria will ensure sustained high predictive accuracy. At an operational level, the online interface allows users to select different functions depending on how well the sequences have been characterized. For example, if the subtype is unknown we have provided an option to select an “unknown” PhenoSeq algorithm that first performs a BLAST align/search using the Los Alamos HIV BLAST tool (default settings) to determine the HIV-1 subtype, and then automatically selects and reports the subtype specific PhenoSeq algorithm used to determine coreceptor usage. To assess the performance of our PhenoSeq BLAST HIV-1 subtyping tool, we tested its accuracy for correctly predicting the HIV-1 subtype of three data sets comprising V3 sequences downloaded from the Los Alamos HIV Database, namely data sets 1, 2 and 3. Each data set consisted of 10 CXCR4-using and 10 R5 V3 sequences from HIV-1 subtypes B, C, D, CRF01_AE, and A or CRF02_AG, totaling 100 discrete V3 sequences per data set. The PhenoSeq “unknown” algorithm correctly predicted the HIV-1 subtype of 93%, 83% and 90% of data sets 1, 2 and 3, respectively, relative to the HIV-1 subtype reported on Los Alamos HIV Database. Currently, PhenoSeq can process up to 10,000 V3 sequences at a time and has been fully integrated with the bulk2clonal software.

AIDS-related deaths have fallen by 30% since 2005 largely due to increased accessibility of antiretroviral therapies to vulnerable HIV-1 affected populations^1^. As a new prognostic tool, PhenoSeq may improve access to maraviroc and future CCR5 blocking drugs, particularly for patients infected with non-B HIV-1 strains who comprise the vast majority of HIV-1 infected individuals worldwide. Furthermore, PhenoSeq may be a valuable tool for the monitoring of novel maraviroc therapies that have advanced to clinical trials, such as its use in intensification therapies to purge viral reservoirs^54^, and as a pre-exposure prophylaxis for the prevention of HIV-1 transmission^55^. In order to maximize the reach and potential public health benefit of PhenoSeq, we have made the platform freely available online at www.burnet.edu.au/phenoseq.

## Methods

### Assembly of phenotypically characterized HIV-1 V3 amino acid sequences

Previously published V3 sequences were obtained from the Los Alamos HIV Database (LANL) (http://www.hiv.lanl.gov/). HIV-1 subtypes were assigned as reported in LANL. Plasma derived bulk V3 sequences were obtained from participants of the Pfizer epidemiology study A4001064^14^,^34^, the Pfizer maraviroc clinical trial MERIT^35^, and from Mulinge *et al*^13^. V3 sequences were defined as “CXCR4-using” if they were documented to solely use CXCR4 (X4) or to use CXCR4 together with CCR5 (R5X4) in phenotypic tropism assays, cause syncytia in MT2 cells or were isolated from plasma virus phenotyped by OTA or ESTA as X4 or dual-mixed. Alternatively, V3 sequences were defined as “R5” if they used CCR5 solely in phenotypic tropism assays, did not cause syncytia in MT2 cells or were isolated from plasma virus phenotyped by OTA or ESTA as R5. We selected one sequence per phenotype per patient using a random number generator to avoid biasing sequence analysis results by resampling related sequences.

In total, from LANL we collected 185 CXCR4-using and 538 R5 HIV-1 subtype B sequences, 80 CXCR4-using and 429 R5 HIV-1 subtype C sequences, 57 CXCR4-using and 80 R5 HIV-1 subtype D sequences, 18 CXCR4-using and 118 R5 HIV-1 subtype A sequences, 41 CXCR4-using and 54 R5 HIV-1 CRF02_AG sequences, and 50 CXCR4-using and 128 R5 HIV-1 CRF01_AE sequences. From the A4001064 study, we obtained bulk V3 nucleotide sequences (one per patient) from 78 subtype A infected individuals (9 CXCR4-using and 69 R5), 95 subtype C infected individuals (55 CXCR4-using and 40 R5) and 87 subtype D infected individuals (43 CXCR4-using and 44 R5), all of which were phenotyping by OTA (Monogram Biosciences). From the MERIT trial, we obtained 615 bulk V3 nucleotide sequences from pre-treatment plasma samples of 205 HIV-1 subtype C infected individuals (18 CXCR4-using and 187 R5), which were phenotyped by ESTA (Monogram Biosciences). From the Mulinge *et al* study^13^, we obtained bulk V3 nucleotide sequences (one per patient) from 53 subtype B infected individuals (12 CXCR4-using and 41 R5), 15 HIV-1 subtype A infected individuals (2 CXCR4-using and 13 R5), 30 CRF02_AG infected individuals (7 CXCR4-using and 23 R5) and 39 CRF01_AE infected individuals (14 CXCR4-using and 25 R5), all of which were phenotyped by an in-house recombinant virus phenotypic tropism assay.

### Sequence analysis parameters

Parameters were used to limit V3 sequence analysis results to alterations most likely to maximize sensitivity for correctly predicting CXCR4-usage, without compromising specificity for correctly predicting R5-tropism. Specifically, cutoffs used to predict CXCR4-usage based on V3 length, charge and/or the number of potential N-linked glycosylation sites were assigned where the frequency in R5 sequences was <5% and the frequency in CXCR4-using sequences was ≥10%. Specific amino acid alterations were considered to have predictive value if the difference in frequency of the alteration between CXCR4-using and R5 sequences was statistically significantly (p < 0.05; two-tailed Fisher's exact t-test) and occurred in ≤10% of CXCR4-using or R5 sequences.

### V3 sequence numbering

Throughout this study V3 sequence amino acids were numbered according to a modified version of the HXB2 V3 sequence numbering system^56^; Cys_1_, Thr_2_, Arg_3_, Pro_4_, Asn_5_, Asn_6_, Asn_7_, Thr_8_, Arg_9_, Lys_10_, Arg_11_, Ile_12_, Arg_13_, Ile_13-14 insert_, Gln_13-14 insert_, Arg_14_, Gly_15_, Pro_16_, Gly_17_, Arg_18_, Ala_19_, Phe_20_, Val_21_, Thr_22_, Ile_23_, Gly_24_, Lys_25_, -_26_, Ile_27_, Gly_28_, Asn_29_, Met_30_, Arg_31_, Gln_32_, Ala_33_, His_34_, Cys_35_.

### Bulk2clonal

At sites of sequence ambiguity, i.e. ≥2 nucleotide variants, bulk2clonal generates all possible nucleotide combinations, based on the International Union of Pure and Applied Chemistry (IUPAC) nomenclature, and translates each possible nucleotide sequence to amino acids. IUPAC nomenclature states that within a nucleotide sequence; R represents the nucleotides A or G, Y represents C or T, S represents G or C, W represents A or T, K represents G or T, M represents A or C, B represents C or G or T, D represents A or G or T, H represents A or C or T, V represents A or C or G and N represents any base.

### Alternative genotypic algorithms

Genotypic algorithms used for comparison were g2p at false positive rates of 1%, 2.5%, 5%, 5.75%, 10%, 15% and 20% (http://coreceptor.bioinf.mpi-inf.mpg.de/)^15^, WebPSSM_X4R5_, WebPSSM_SINSI_ and subtype C specific WebPSSM_SINSI_-C, using default cutoff settings (http://indra.mullins.microbiol.washington.edu/webpssm/)^31^,^32^, HIVcoPRED split amino acid compositions (SAAC) and SAAC + Basic Local Alignment Search Tool (SAAC + BLAST) algorithms (http://www.imtech.res.in/raghava/hivcopred/submit.html) at default thresholds^50^, dsKernel (http://www.webcitation.org/query.php?url=http://genome.ulaval.ca/hiv-dskernel&refdoi=10.1186/1742-4690-5-110)^57^, the 11/25 rule, the 11 and/or 25 rule, the 11/24/25 rule^30^,^58^, the Raymond *et al* 11/25 + V3 charge rule^22^,^43^, the Lin *et al* rule^59^, the Raymond *et al* subtype D specific rules^23^, the Raymond *et al* CRF01_AE specific rules^39^ and the Esbjömsson *et al* subtype A and CRF02_AG specific rules^60^.

## Supplementary Material

Supplementary Information Supplementary material

## Figures and Tables

**Table 1:** Sensitivity, specificity and AUROC of genotypic algorithms for predicting coreceptor usage of training set V3 sequences

		PhenoSeq	g2p FPR 5.75%	g2p FPR 10%	WebPSSM_X4R5_	WebPSSM_SI/NSI_*
**PhenoSeq-B**	**Sens/Spec**	80.7/82.9	68.8/93.7	75.3/86.6	53.8/95.8	61.3/94.7
**Training Set**	**AUROC**	0.82	0.81 (p = 0.40)	0.81 (p = 0.40)	0.75 (p = 0.05)	0.78 (p = 0.17)
93 CXCR4-using, 269 R5						
**PhenoSeq-C**	**Sens/Spec**	88.8/87.9	75/96.5	81.3/93.7	-	82.5/89
**Training Set**	**AUROC**	0.88	0.86 (p = 0.24)	0.88 (p = 0.40)	-	0.86 (p = 0.24)
80 CXCR4-using, 429 R5						
**PhenoSeq-D**	**Sens/Spec**	87.8/87.5	81.4/72.7	88.4/61.4	81.4/65.9	69.8/63.6
**Training Set**	**AUROC**	0.88	0.77 (***p = 0.02***)	0.72 (***p < 0.01***)	0.72 (***p = 0.03***)	0.77 (***p = 0.02***)
57 CXCR4-using, 80 R5						
**PhenoSeq-AE**	**Sens/Spec**	88/92.2	82/76.6	88/56.3	78/78.9	80/81.3
**Training Set**	**AUROC**	0.90	0.79 (***p = 0.02***)	0.72 (***p < 0.01***)	0.79 (***p < 0.01***)	0.81 (***p = 0.03***)
50 CXCR4-using, 128 R5						
**PhenoSeq-A/AG**	**Sens/Spec**	59.7/87.1	29/96	40.3/91.5	27.4/92	29/97
**Training Set**	**AUROC**	0.73	0.63 (***p = 0.03***)	0.659 (p = 0.10)	0.60 (***p < 0.01***)	0.63 (***p = 0.04***)
59 CXCR4-using, 172 R5						

Sens, % sensitivity for correctly predicting CXCR4-usage (relative to phenotypic tropism assay results) was calculated by dividing the number of correctly predicted CXCR4-using sequences by the total number CXCR4-using sequences and multiplying by 100. Spec, % specificity for correctly predicting R5 strains (relative to phenotypic tropism assay results) was calculated by dividing the number of correctly predicted R5 sequences by the total number of R5 sequences and multiplying by 100. P-values (two-tailed) for comparing area under the receiver operator characteristic curves (AUROC) was performed according to Hanley *et al*^33^. P-values < 0.05 were considered significant and are highlighted in bold italicized text. FPR, false positive rate.

*The subtype C specific WebPSSM_SI/NSI_ algorithm was used for subtype C HIV-1 predictions.

**Table 2:** Sensitivity, specificity and AUROC of genotypic algorithms for predicting coreceptor usage of test set V3 sequences

		PhenoSeq	g2p FPR 5.75%	g2p FPR 10%	WebPSSM_X4R5_	WebPSSM_SI/NSI_*
**PhenoSeq-B Test Set 1**	**Sens/Spec**	100/87.8	100/97.6	100/90.2	41.7/95.1	16.7/87.8
Mulinge *et al*	**AUROC**	0.94	0.99 (p = 0.17)	0.95 (p = 0.44)	0.68 (***p < 0.01***)	0.52 (***p < 0.01***)
12 CXCR4-using, 41 R5						
**PhenoSeq-B Test Set 2**	**Sens/Spec**	78.4/80.3	70.7/92.2	72.8/84	55.4/96.3	62/94.1
Los Alamos HIV Database	**AUROC**	0.79	0.82 (p = 0.32)	0.78 (p = 0.41)	0.76 (p = 0.24)	0.78 (p = 0.41)
92 CXCR4-using, 269 R5						
**PhenoSeq-C Test Set 1**	**Sens/Spec**	83.6/92.5	78.2/95	81.8/95	-	85.5/77.5
A4001064	**AUROC**	0.88	0.87 (p = 0.22)	0.88 (p = 0.30)	-	0.82 (p = 0.22)
55 CXCR4-using, 40 R5						
**PhenoSeq-C Test Set 2**	**Sens/Spec**	77.8/75.4	11.1/93	50/82.4	-	61.1/81.3
MERIT	**AUROC**	0.77	0.52 (***p < 0.01***)	0.66 (p = 0.15)	-	0.71 (p = 0.29)
18 CXCR4-using, 187 R5						
**PhenoSeq-D Test Set**	**Sens/Spec**	80.5/77.3	81.4/72.7	88.4/61.4	81.4/65.9	69.8/63.6
A4001064	**AUROC**	0.79	0.77 (p = 0.40)	0.75 (p = 0.29)	0.74 (p = 0.24)	0.67 (p = 0.05)
43 CXCR4-using, 44 R5						
**PhenoSeq-AE Test Set**	**Sens/Spec**	85.7/96	78.6/68	85.7/56	85.7/72	85.7/76
A4001064	**AUROC**	0.91	0.73 (***p = 0.046***)	0.71 (***p = 0.03***)	0.79 (p = 0.11)	0.81 (p = 0.15)
14 CXCR4-using, 25 R5						
**PhenoSeq-A/AG Test Set 1**	**Sens/Spec**	88.9/76.8	44.4/97.1	55.6/94.2	44.4/87	66.7/84
A4001064	**AUROC**	0.83	0.71 (p = 0.18)	0.75 (p = 0.27)	0.66 (p = 0.10)	0.75 (p = 0.28)
9 CXCR4-using, 69 R5						
**PhenoSeq-A/AG Test Set 2**	**Sens/Spec**	62.5/97.2	50/94.4	62.5/80.6	37.5/88.9	37.5/91.7
Mulinge *et al*	**AUROC**	0.80	0.72 (p = 0.30)	0.72 (p = 0.29)	0.63 (p = 0.14)	0.65 (p = 0.16)
8 CXCR4-using, 36 R5						

Sens (% sensitivity) and Spec (% specificity) was calculated as described in the Table 1 legend. P-values (two-tailed) for comparisons of area under the receiver operator characteristic curve (AUROC) were performed according to Hanley *et al*^33^. P-values < 0.05 were considered significant and are highlighted in bold italicized text. FPR, false positive rate.

*The subtype C specific WebPSSM_SI/NSI_ algorithm was used for subtype C HIV-1 predictions.
