# Supplementary material for: Reliable Genotypic Tropism Tests for the Major HIV-1 Subtypes
Source: Sci Rep. 2015 Feb 25;5:8543. doi: 10.1038/srep08543 (PMC4894445; doi:10.1038/srep08543)
Supplement: Supplementary Information — Supplementary material [file srep08543-s1.pdf]

Supplementary Material

Reliable Genotypic Tropism Tests for the Major HIV-1 Subtypes

Kieran Cashin [1,2], Lachlan R. Gray [1,3], Katherine L. Harvey [1,2], Danielle Perez-Bercoff [4], Guinevere Q. Lee [5], Jasminka Sterjovski [1,3], Michael Roche [1,3], James F. Demarest [6], Fraser Drummond [7], P. Richard Harrigan [5], Melissa J. Churchill [1,8,9] and Paul R. Gorry [1,2,3,10*].

[1] Center for Biomedical Research, Burnet Institute, Melbourne, Australia 3004;

[2] Department of Microbiology and Immunology, University of Melbourne, Parkville, Australia 3010;

[3] Department of Infectious Diseases, Monash University, Melbourne, Australia 3800;

[4] Laboratory of Retrovirology, Centre Recherche Public de la Santé, Luxembourg 1526;

[5] BC Centre for Excellence in HIV/AIDS, Vancouver, Canada Y6Z 1Y6;

[6] ViiV Healthcare, Research Triangle Park, North Carolina, USA 27709-3398;

[7] ViiV Healthcare Australia, Abbotsford, Australia 3067;

[8] Department of Medicine, Monash University, Melbourne, Australia 3800;

[9] Department of Microbiology, Monash University, Melbourne, Australia 3800.

[10] School of Applied Sciences, College of Science, Engineering and Health, RMIT University, Melbourne, Australia 3001.

[*] ***Corresponding author***: Paul R. Gorry, College of Science, Engineering and Health, RMIT University, Melbourne, Australia 3001.

Phone: +61-3-9925-0993; Email: paul.gorry@rmit.edu.au

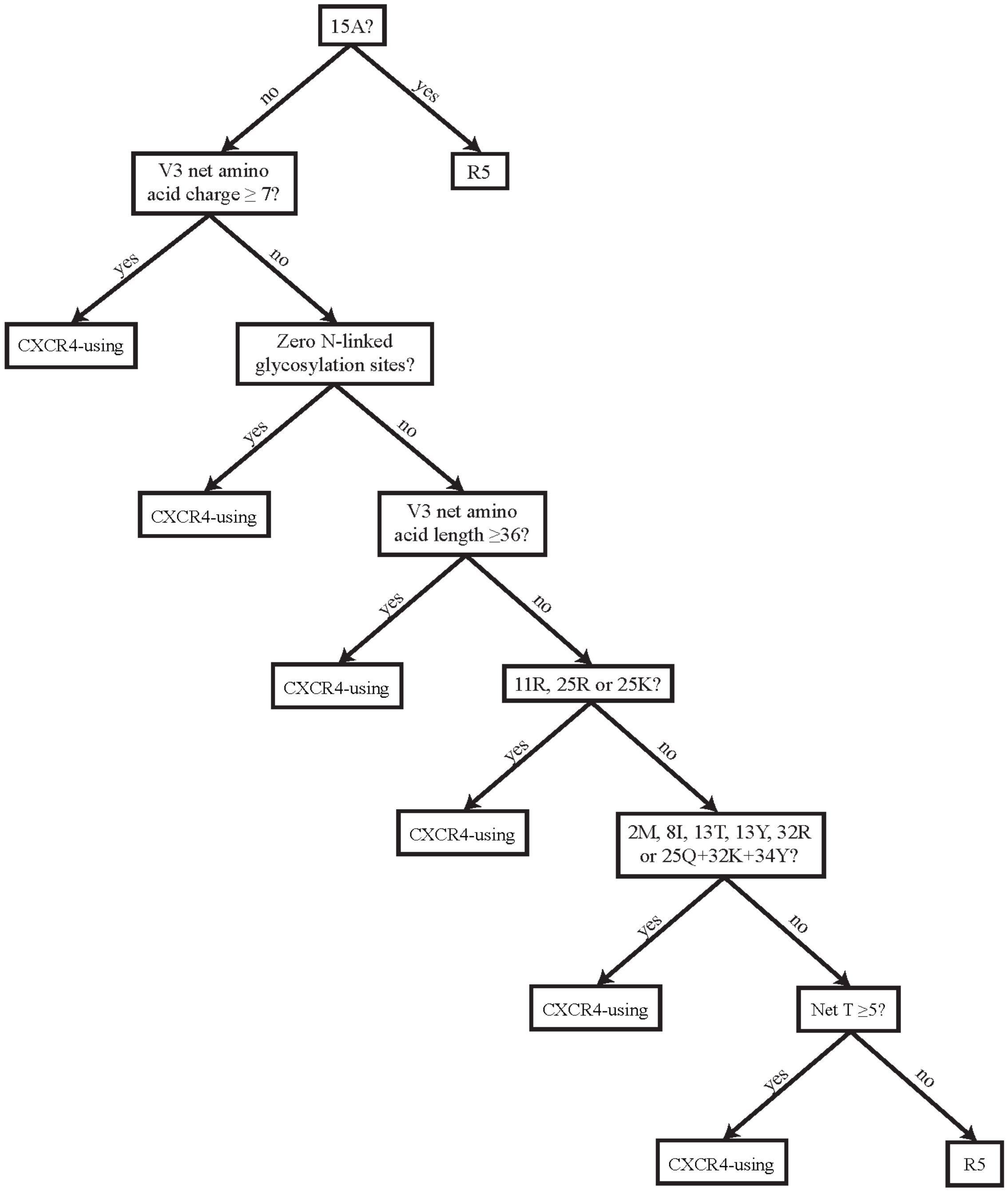

Supplementary Figure 1. Diagrammatic representation of PhenoSeq-B.

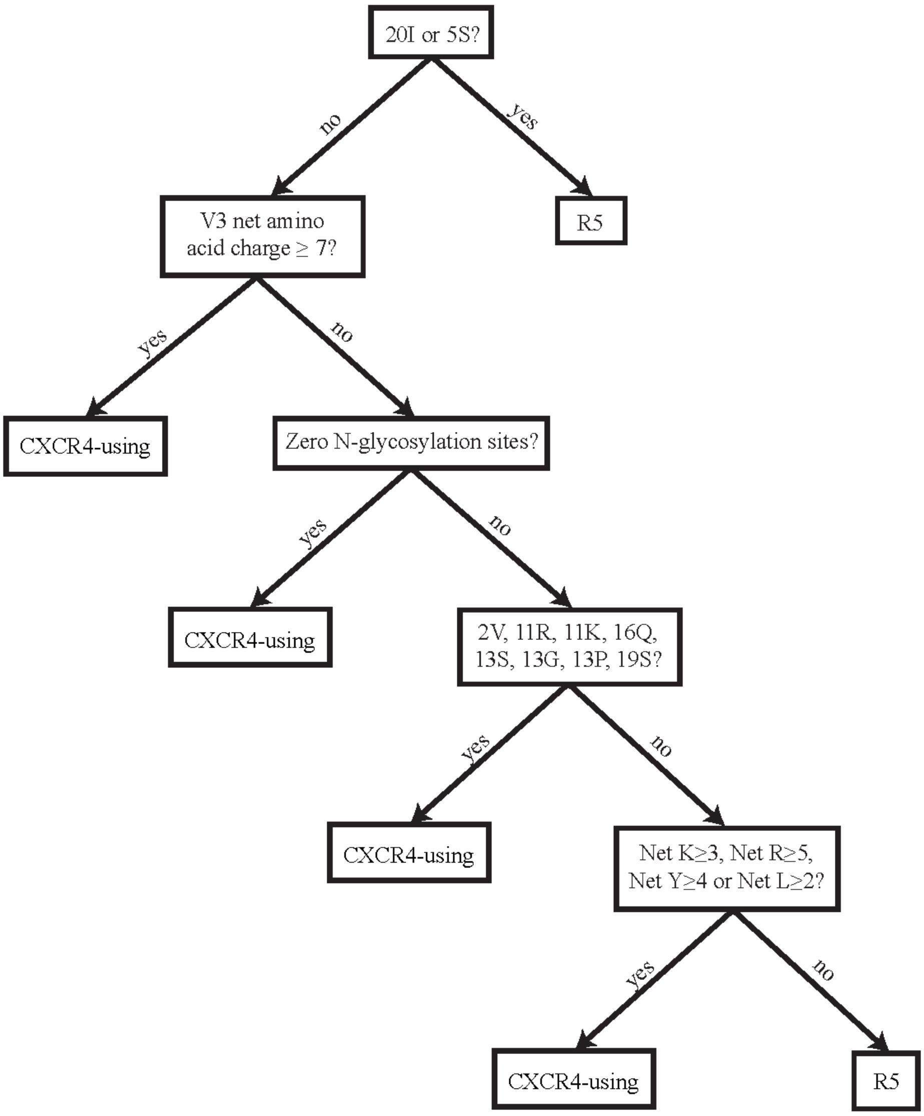

Supplementary Figure 2. Diagrammatic representation of PhenoSeq-D.

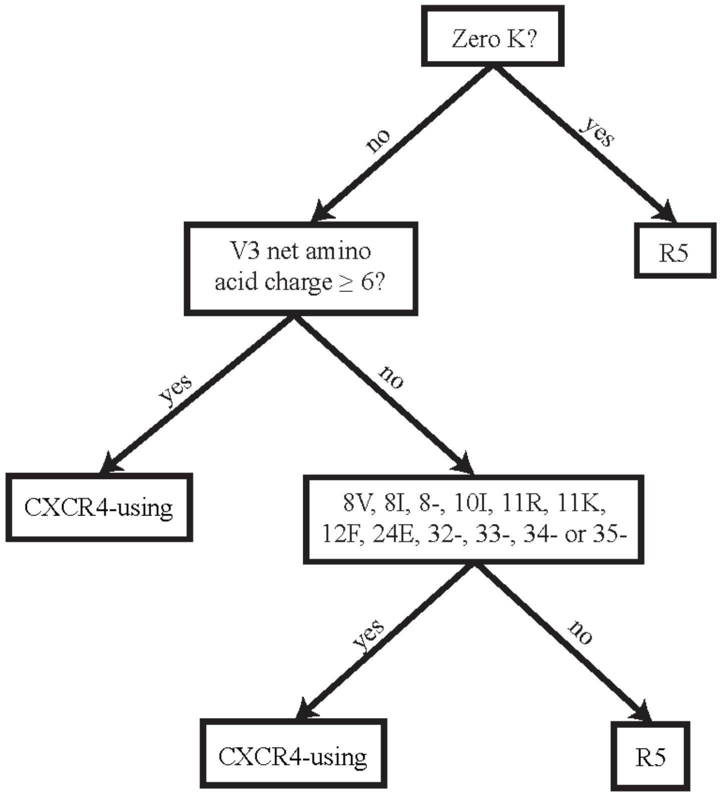

Supplementary Figure 3. Diagrammatic representation of PhenoSeq-AE.

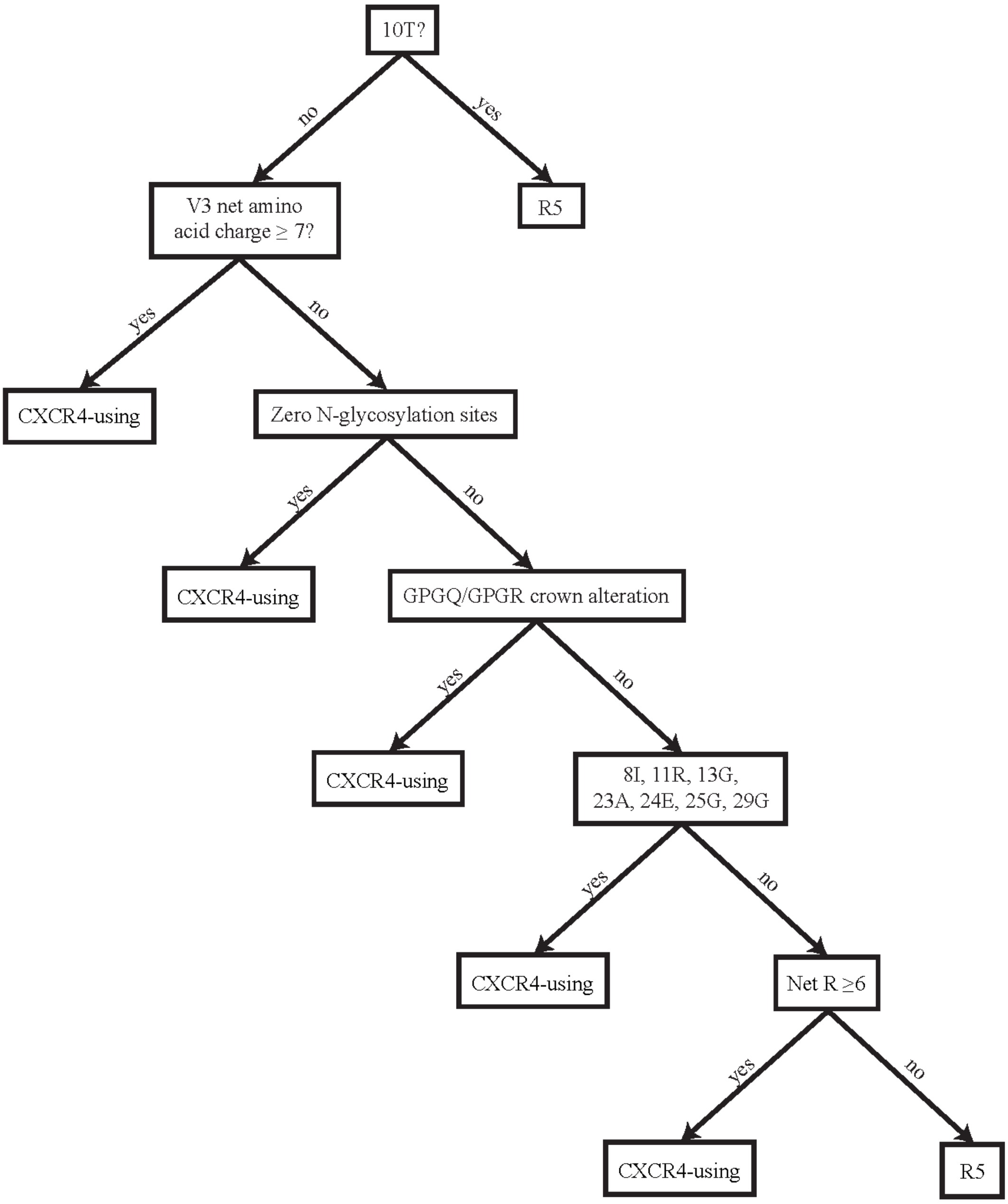

Supplementary Figure 4. Diagrammatic representation of PhenoSeq-A/AG

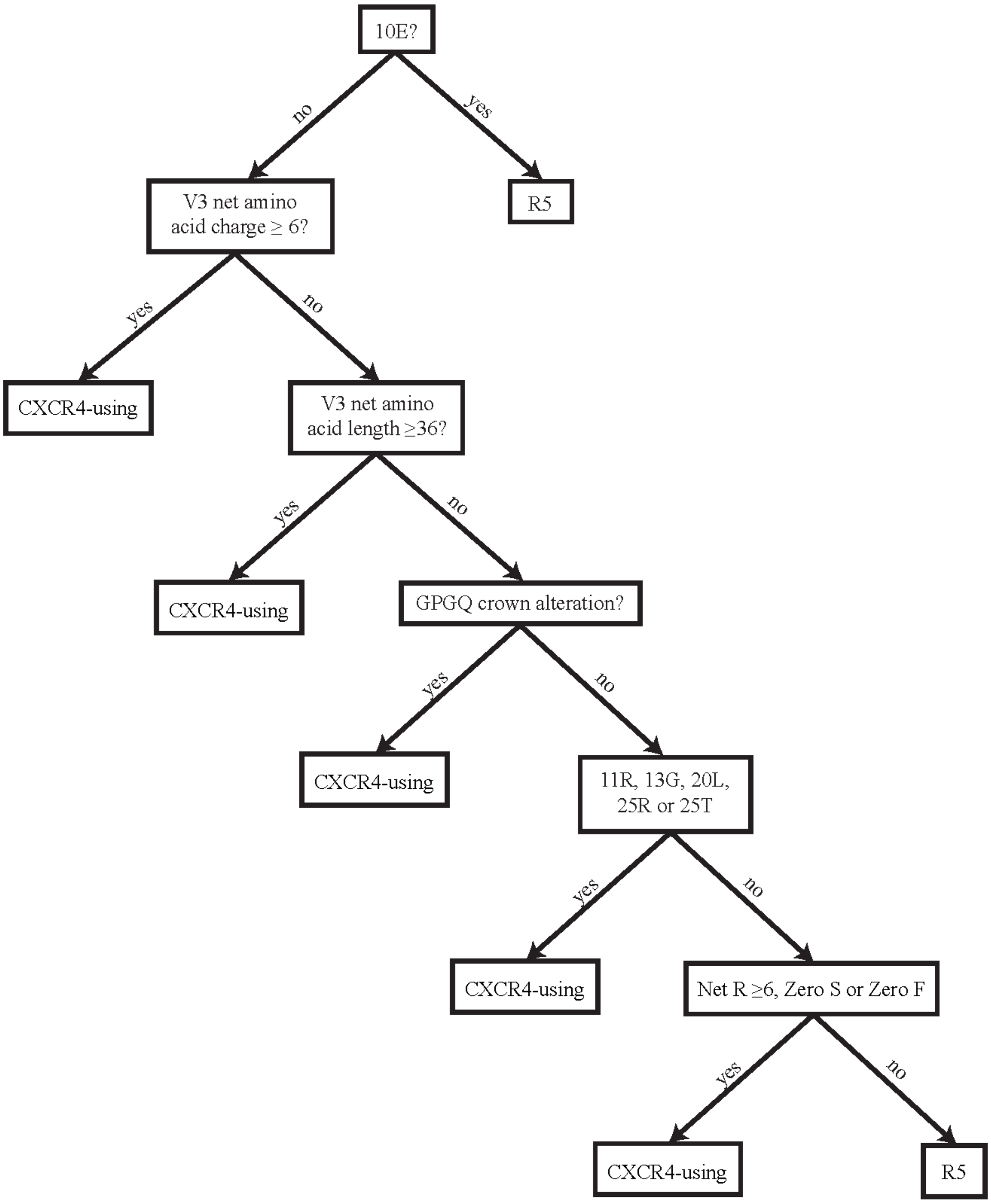

Supplementary Figure 5. Diagrammatic representation of PhenoSeq-C

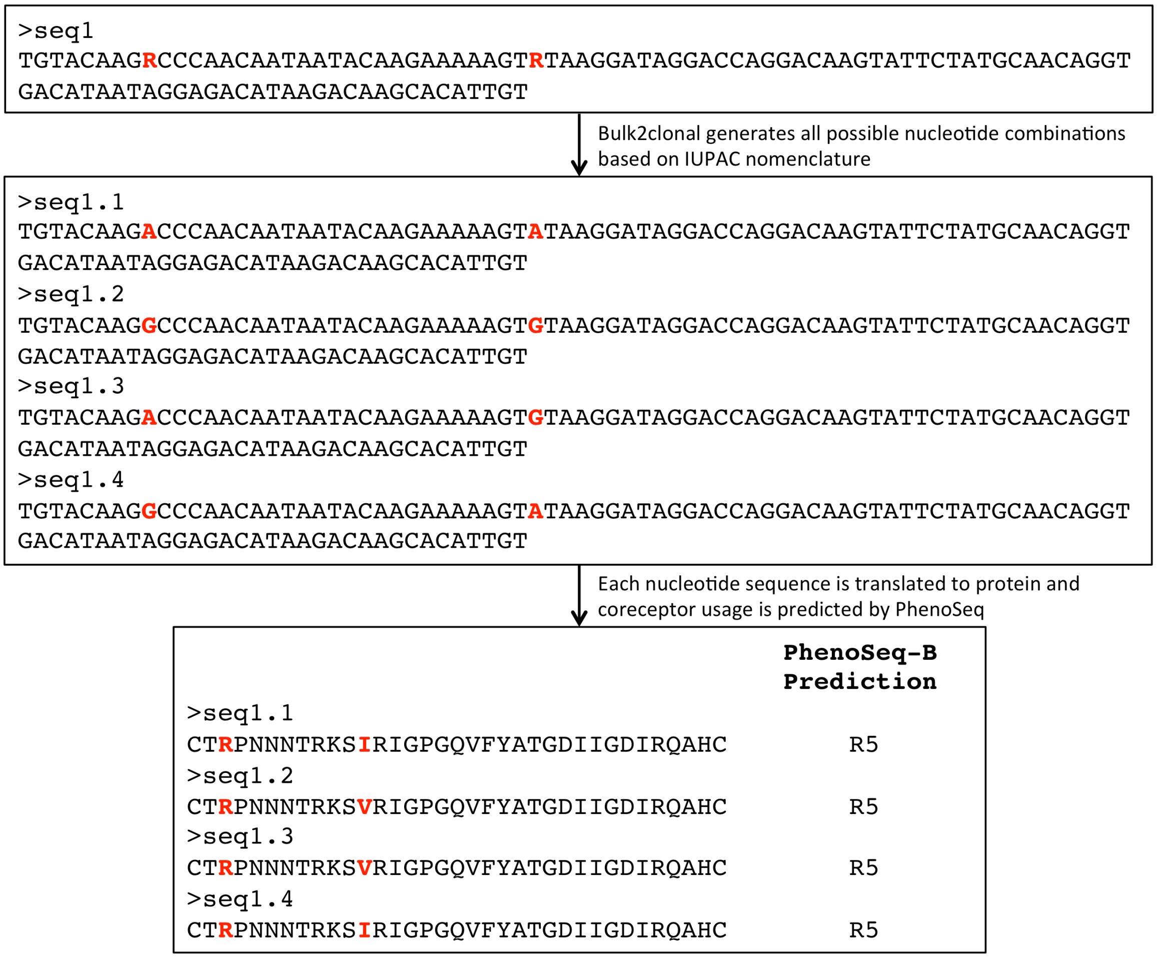

Supplementary Figure 6. Illustration of bulk2clonal functionality.

Supplementary Table 1. Summary of sensitivity, specificity and AUROC of genotypic algorithms for predicting coreceptor usage of subtype B V3 sequences.

	**Training Set** Los Alamos HIV Database 93 CXCR4-using, 269 R5					**Test Set 1** Mulinge *et al* 12 CXCR4-using, 41 R5					**Test Set 2** Los Alamos HIV Database 92 CXCR4-using, 269 R5				
	**Sens**	**Spec**	**PPV**	**NPV**	**AUROC**	**Sens**	**Spec**	**PPV**	**NPV**	**AUROC**	**Sens**	**Spec**	**PPV**	**NPV**	**AUROC**
PhenoSeq-B	80.7	82.9	62	92.6	0.82	100	87.8	70.6	100	0.94	78.4	80.3	57.6	91.6	0.79
g2p FPR 1%	37.6	99.3	94.9	82.2	0.69 (***p<0.01***)	25	100	100	82	0.56 (***p<0.01***)	33.7	99.3	94.3	81.4	0.67 (***p<0.01***)
g2p FPR 2.5%	58.1	96.7	85.9	87	0.77 (p=0.12)	33.3	100	100	83.7	0.58 (***p<0.01***)	58.7	97.8	90.1	87.4	0.78 (p=0.41)
g2p FPR 5%	68.8	94.1	80.1	89.7	0.82 (p=0.40)	100	97.6	92.4	100	0.99 (p=0.17)	70.7	92.9	77.3	90.3	0.82 (p=0.23)
g2p FPR 5.75%	68.8	93.7	79.1	89.7	0.81 (p=0.40)	100	97.6	92.4	100	0.99 (p=0.17)	70.7	92.2	75.6	90.2	0.82 (p=0.32)
g2p FPR 10%	75.3	86.6	66	91	0.81 (p=0.40)	100	90.2	74.9	100	0.95 (p=0.44)	72.8	84	60.9	90	0.78 (p=0.41)
g2p FPR 15%	76.3	79.2	55.9	90.6	0.78 (p=0.17)	100	85.4	66.7	100	0.93 (p=0.44)	72.8	80.3	55.8	89.6	0.77 (p=0.32)
g2p FPR 20%	78.5	67.3	45.4	90.1	0.73 (***p=0.02***)	100	82.9	63.1	100	0.92 (p=0.35)	77.2	72.5	49	90.3	0.75 (p=0.18)
$WebPSSM_{X4R5}$	53.8	95.8	81.6	85.7	0.75 (p=0.05)	41.7	95.1	71.4	84.8	0.68 (***p<0.01***)	55.4	96.3	83.7	86.3	0.76 (p=0.24)
$WebPSSM_{SI/NSI}$	61.3	94.7	80	87.6	0.78 (p=0.17)	16.7	87.8	28.6	78.3	0.52 (***p<0.01***)	62	94.1	78.2	87.9	0.78 (p=0.41)
11/25	55.9	93.3	74.3	86	0.75 (p=0.05)	41.7	92.7	62.6	84.5	0.67 (***p<0.01***)	50	92.6	69.8	84.4	0.71 (***p=0.04***)
11/24/25	58.1	92.9	73.9	86.5	0.76 (p=0.08)	41.7	92.7	62.6	84.5	0.67 (***p<0.01***)	52.2	91.4	67.5	84.8	0.72 (p=0.06)
11/25+V3 charge	58.1	93.7	76.1	86.6	0.76 (p=0.08)	58.3	92.7	70	88.4	0.76 (***p=0.04***)	54.3	94.4	76.8	85.8	0.74 (p=0.13)
HIVcoPRED (SAAC)	67.7	94.4	80.7	89.4	0.82 (p=0.40)	83.3	90.2	71.3	94.9	0.87 (p=0.20)	69.6	94.8	82.1	90.1	0.82 (p=0.23)
HIVcoPRED (SAAC+BLAST)	68.8	94.1	80.1	89.7	0.75 (p=0.05)	83.3	90.2	71.3	94.9	0.87 (p=0.20)	71.7	94.8	82.5	90.7	0.83 (p=0.16)
dsKernel	53.8	96.3	83.4	85.8	0.75 (p=0.05)	100	34.1	30.8	100	0.67 (***p<0.01***)	55.4	94.4	77.2	86.1	0.75 (p=0.18)

Sens, % sensitivity for correctly predicting CXCR4-usage (relative to phenotypic tropism assay results) was calculated by dividing the number of correctly predicted CXCR4-using sequences by the total number of CXCR4-using sequences, then multiplying by 100. Spec, % specificity for correctly predicting R5 strains (relative to phenotypic tropism assay results) was calculated by dividing the number of correctly predicted R5 sequences by the total number of R5 sequences, then multiplying by 100. PPV, % positive predictive value was calculated by dividing the number of correctly predicted CXCR4-using sequence by the total number of V3 sequences predicted to be CXCR4-using, then multiplying by 100. NPV, % negative predictive value was calculated by dividing the number of correctly predicted R5 sequence by the total number of V3 sequences predicted to be R5, then multiplying by 100. P-values (two-tailed) for comparing area under the receiver operator characteristic curves (AUROC) was performed according to Hanley *et al*[1]. P-values <0.05 were considered significant and are highlighted in bold italicized text. FPR, false positive rate.

Supplementary Table 2. Summary of sensitivity, specificity and AUROC of genotypic algorithms for predicting coreceptor usage of subtype D V3 sequences.

	**Training Set**<br>Los Alamos HIV Database<br>57 CXCR4-using, 80 R5					**Test Set**<br>A4001064<br>43 CXCR4-using, 44 R5				
	**Sens**	**Spec**	**PPV**	**NPV**	**AUROC**	**Sens**	**Spec**	**PPV**	**NPV**	**AUROC**
PhenoSeq-D	87.7	87.5	83.3	90.9	0.88	80.5	77.3	77.6	80.2	0.79
g2p FPR 1%	49.1	97.5	93.3	72.9	0.73 (***p<0.01***)	51.2	88.6	81.4	65	0.56 (***p<0.01***)
g2p FPR 2.5%	70.2	87.5	80	80.5	0.79 (***p=0.046***)	79.1	72.7	73.9	78.1	0.70 (p=0.06)
g2p FPR 5%	86	70	67.1	87.5	0.78 (***p=0.03***)	81.4	72.7	74.5	80	0.76 (p=0.34)
g2p FPR 5.75%	86	67.5	65.3	87.1	0.77 (***p=0.02***)	88.4	61.4	69.1	84.4	0.77 (p=0.40)
g2p FPR 10%	89.5	53.8	58	87.8	0.72 (***p<0.01***)	90.7	59.1	68.4	86.7	0.75 (p=0.29)
g2p FPR 15%	89.5	52.5	57.3	87.5	0.71 (***p<0.01***)	90.7	54.5	66.1	85.7	0.75 (p=0.29)
g2p FPR 20%	89.5	48.8	55.5	86.7	0.69 (***p<0.01***)	81.4	65.9	70	78.4	0.73 (p=0.19)
$\text{WebPSSM}_{\text{X4R5}}$	89.5	55	58.6	88	0.72 (***p=0.03***)	69.8	63.6	65.2	68.3	0.74 (p=0.24)
$\text{WebPSSM}_{\text{SI/NSI}}$	86	67.5	65.3	87.1	0.77 (***p=0.02***)	34.9	84.1	68.2	56.9	0.67 (p=0.05)
11/25	78.9	63.8	60.8	80.9	0.71 (***p<0.01***)	34.9	81.8	65.2	56.3	0.60 (***p<0.01***)
11/24/25	78.9	61.3	59.2	80.3	0.70 (***p<0.01***)	48.8	81.8	72.4	62	0.58 (***p<0.01***)
11/25+V3 charge	68.4	70	61.9	75.7	0.69 (***p<0.01***)	51.2	81.8	73.3	63.2	0.65 (***p<0.01***)
Raymond *et al* D-HIV	50.9	95	87.9	73.1	0.73 (***p<0.01***)	48.8	95.5	91.4	65.6	0.72 (p=0.05)
HIVcoPRED (SAAC)	82.5	86.3	81.1	87.4	0.84 (p=0.25)	88.4	31.8	55.9	73.7	0.60 (***p<0.01***)
HIVcoPRED (SAAC+BLAST)	82.5	86.3	81.1	87.4	0.84 (p=0.25)	88.4	31.8	55.9	73.7	0.60 (***p<0.01***)

Sens (% sensitivity), Spec (% specificity), PPV (% positive predictive value) and NPV (% negative predictive value) were calculated as described in Supplementary Table 1 legend. P-values (two-tailed) of the difference in the area under the receiver operator characteristic curves (AUROC) between PhenoSeq and the indicated alternate genotypic algorithm are shown in parenthesis, calculated as described by Hanley *et al*[1]. P-values <0.05 were considered significant and are highlighted in bold italicized text. FPR, false positive rate.

Supplementary Table 3. Summary of sensitivity, specificity and AUROC of genotypic algorithms for predicting coreceptor usage of subtype CRF01_AE V3 sequences.

	**Training Set** Los Alamos HIV Database 50 CXCR4-using, 128 R5					**Test Set** Mulinge *et al* 14 CXCR4-using, 25 R5				
	**Sens**	**Spec**	**PPV**	**NPV**	**AUROC**	**Sens**	**Spec**	**PPV**	**NPV**	**AUROC**
PhenoSeq-AE	88	92.2	81.5	95.2	0.90	85.7	96	92.3	92.3	0.91
g2p FPR 1%	60	97.7	91.1	86.2	0.79 (***p<0.01***)	21.4	100	100	69.4	0.55 (***p<0.01***)
g2p FPR 2.5%	80	89.8	75.4	92	0.85 (p=0.14)	64.3	92	81.8	82.1	0.78 (p=0.10)
g2p FPR 5%	82	79.7	61.2	91.9	0.81 (***p=0.03***)	78.6	80	68.8	87	0.79 (p=0.12)
g2p FPR 5.75%	82	76.6	57.8	91.6	0.79 (***p=0.02***)	78.6	68	57.9	85	0.73 (***p=0.046***)
g2p FPR 10%	88	56.3	44	92.3	0.72 (***p<0.01***)	85.7	56	52.2	87.5	0.71 (***p=0.03***)
g2p FPR 15%	88	50	40.7	91.4	0.69 (***p<0.01***)	92.9	44	48.2	91.7	0.68 (***p=0.02***)
g2p FPR 20%	90	39.8	36.9	91.1	0.65 (***p<0.01***)	92.9	44	48.2	91.7	0.68 (***p=0.02***)
WebPSSM$_{X4R5}$	78	78.9	59.1	90.2	0.79 (***p<0.01***)	85.7	72	63.2	90	0.79 (p=0.11)
WebPSSM$_{SI/NSI}$	80	81.3	62.6	91.2	0.81 (***p=0.03***)	85.7	76	66.7	90.5	0.81 (p=0.15)
11/25	50	97.7	89.5	83.3	0.74 (***p<0.01***)	7.1	100	100	65.8	0.52 (***p<0.01***)
11/24/25	52	97.7	89.8	83.9	0.75 (***p<0.01***)	21.4	100	100	69.4	0.55 (***p<0.01***)
11/25+V3 charge	54	97.7	90.2	84.5	0.76 (***p<0.01***)	21.4	100	100	69.4	0.55 (***p<0.01***)
Raymond *et al* AE-HIV	72	93	80.1	89.5	0.83 (p=0.06)	71.4	96	90.9	85.7	0.84 (p=0.22)
HIVcoPRED (SAAC)	88	85.2	69.9	94.8	0.87 (p=0.22)	85.7	92	85.7	92	0.89 (p=0.41)
HIVcoPRED (SAAC+BLAST)	90	85.2	70.4	95.6	0.88 (p=0.29)	85.7	92	85.7	92	0.89 (p=0.41)
dsKernel	92	74.2	58.2	96	0.83 (***p=0.07***)	78.6	88	78.6	88	0.83 (p=0.21)

Sens (% sensitivity), Spec (% specificity), PPV (% positive predictive value) and NPV (% negative predictive value) were calculated as described in Supplementary Table 1 legend. P-values (two-tailed) of the difference in the area under the receiver operator characteristic curves (AUROC) between PhenoSeq and the indicated alternate genotypic algorithm are shown in parenthesis, calculated as described by Hanley *et al*[1]. P-values <0.05 were considered significant and are highlighted in bold italicized text. FPR, false positive rate.

Supplementary Table 4. Summary of sensitivity, specificity and AUROC of genotypic algorithms for predicting coreceptor usage of subtype A and CRF01_AG V3 sequences.

	**Training Set** Los Alamos HIV Database 59 CXCR4-using, 172 R5					**Test Set 1** A4001064 9 CXCR4-using, 69 R5					**Test Set 2** Mulinge *et al* 8 CXCR4-using, 36 R5				
	**Sens**	**Spec**	**PPV**	**NPV**	**AUROC**	**Sens**	**Spec**	**PPV**	**NPV**	**AUROC**	**Sens**	**Spec**	**PPV**	**NPV**	**AUROC**
PhenoSeq-A/AG	59.7	87.1	61.4	86.3	0.73	88.9	76.8	33.3	98.1	0.83	62.5	97.2	83.2	92.1	0.80
g2p FPR 1%	16.1	100	100	77.7	0.54 (***p<0.01***)	0	100	0	88.5	0.50 ***(p=0.01)***	12.5	100	100	83.7	0.53 (***p=0.04***)
g2p FPR 2.5%	25.8	98.5	85.5	79.5	0.62 (***p=0.03***)	22.2	98.6	67.4	90.7	0.60 ***(p=0.048)***	37.5	100	100	87.8	0.59 (p=0.09)
g2p FPR 5%	29	96.5	74	79.8	0.63 (***p=0.03***)	44.4	97.1	66.6	93.1	0.71 (p=0.18)	37.5	94.4	59.8	87.2	0.66 (p=0.18)
g2p FPR 5.75%	29	96	71.3	79.8	0.63 (***p=0.03***)	44.4	97.1	66.6	93.1	0.71 (p=0.18)	50	94.4	66.5	89.5	0.72 (p=0.30)
g2p FPR 10%	40.3	91.5	61.9	81.7	0.66 (p=0.10)	55.6	94.2	55.6	94.2	0.75 (p=0.27)	62.5	80.6	41.7	90.6	0.72 (p=0.29)
g2p FPR 15%	50	87.1	57.1	83.5	0.69 (p=0.20)	66.7	89.9	46.3	95.4	0.78 (p=0.36)	75	72.2	37.5	92.9	0.74 (p=0.34)
g2p FPR 20%	50	85.6	54.4	83.3	0.68 (p=0.16)	66.7	87	40.1	95.2	0.77 (p=0.32)	75	72.2	37.5	92.9	0.74 (p=0.34)
WebPSSM$_{X4R5}$	27.4	92	54	78.7	0.60 (***p<0.01***)	44.4	87	30.8	92.3	0.66 (p=0.10)	37.5	88.9	42.9	86.5	0.63 (p=0.14)
WebPSSM$_{SI/NSI}$	29	97	76.8	79.9	0.63 (***p=0.04***)	66.7	84	35.2	95.1	0.75 (p=0.28)	37.5	91.7	50.1	86.8	0.65 (p=0.16)
11/25	17.7	97	66.9	77.5	0.57 (***p<0.01***)	22.2	95.7	40.2	90.4	0.59 ***(p=0.04)***	12.5	100	100	83.7	0.53 (***p=0.04***)
11/24/25	21	96.5	67.3	78.1	0.59 (***p<0.01***)	22.2	95.7	40.2	90.4	0.59 ***(p=0.04)***	12.5	100	100	83.7	0.53 (***p=0.04***)
11/25+V3 charge	19.4	97.5	72.7	77.9	0.59 (***p<0.01***)	33.3	95.7	50.3	91.7	0.65 (p=0.09)	25	100	100	85.7	0.56 (p=0.06)
HIVcoPRED (SAAC)	80.6	92	77.6	93.3	0.86 (***p<0.01***)	88.9	56.5	21	97.5	0.73 (p=0.22)	50	66.7	25	85.7	0.58 (p=0.08)
HIVcoPRED (SAAC+BLAST)	85.5	92	78.6	94.9	0.89 (***p<0.01***)	88.9	56.5	21	97.5	0.73 (p=0.22)	50	66.7	25	85.7	0.58 (p=0.08)
HIVcoPRED (SAAC)+*bulk2clonal*	85.5	92	78.6	94.9	0.89 (***p<0.01***)	77.8	63.8	21.9	95.7	0.71 (p=0.18)	62.5	72.2	33.3	89.7	0.67 (p=0.20)
dsKernel	24.2	98	80.6	79	0.61 (***p=0.02***)	33.3	94.2	42.8	91.5	0.64 (p=0.08)	50	66.7	25	85.7	0.58 (p=0.08)
Net ≥4	46.8	59.2	28.2	76.4	0.53 (***p<0.01***)	44.4	68.1	15.4	90.4	0.56 ***(p=0.03)***	37.5	63.9	18.8	82.1	0.51 (***p=0.03***)
Net ≥5	30.6	90.5	52.5	79.2	0.61 (***p<0.01***)	11.1	91.3	14.3	88.7	0.51 ***(p=0.01)***	25	86.1	28.6	83.8	0.56 (p=0.06)
Total ≥8	21	84.1	31.2	75.6	0.53 (***p<0.01***)	11.1	95.7	25.2	89.2	0.53 ***(p=0.01)***	37.5	88.9	42.9	86.5	0.63 (p=0.14)
Net ≥5 and Total ≥8	37.1	98.5	89.5	82	0.68 (p=0.16)	22.2	88.4	20	89.7	0.55 ***(p=0.02)***	37.5	77.8	27.3	84.9	0.58 (p=0.07)

Sens (% sensitivity), Spec (% specificity), PPV (% positive predictive value) and NPV (% negative predictive value) were calculated as described in Supplementary Table 1 legend. P-values (two-tailed) of the difference in the area under the receiver operator characteristic curves (AUROC) between PhenoSeq and the indicated alternate genotypic algorithm are shown in parenthesis, calculated as described by Hanley *et al*[1]. P-values <0.05 were considered significant and are highlighted in bold italicized text. FPR, false positive rate.

Supplementary Table 5. Summary of sensitivity, specificity and AUROC of genotypic algorithms for predicting coreceptor usage of subtype C V3 sequences.

	**Training Set** Los Alamos HIV Database 80 CXCR4-using, 429 R5					**Test Set 1** A4001064 55 CXCR4-using, 40 R5					**Test Set 2** MERIT 18 CXCR4-using, 187 R5				
	**Sens**	**Spec**	**PPV**	**NPV**	**AUROC**	**Sens**	**Spec**	**PPV**	**NPV**	**AUROC**	**Sens**	**Spec**	**PPV**	**NPV**	**AUROC**
PhenoSeq-C	88.8	87.9	57.8	97.7	0.88	83.6	92.5	93.9	80.4	0.88	77.8	75.4	23.3	97.2	0.77
g2p FPR 1%	52.6	99.8	98	91.9	0.76 (***p<0.01***)	32.7	97.5	94.7	51.3	0.65 (***p=0.03***)	0	100	0	91.2	0.50 (***p<0.01***)
g2p FPR 2.5%	68.8	99.8	98.5	94.5	0.84 (p=0.14)	58.2	97.5	97	62.9	0.78 (p=0.17)	5.6	98.9	32.9	91.6	0.52 (***p<0.01***)
g2p FPR 5%	73.8	96.5	79.7	95.2	0.85 (p=0.20)	76.4	95	95.5	74.5	0.86 (p=0.19)	5.6	93	7.1	91.1	0.49 (***p<0.01***)
g2p FPR 5.75%	75	96.5	80	95.4	0.86 (p=0.24)	78.2	95	95.6	76	0.87 (p=0.22)	11.1	93	13.2	91.6	0.52 (***p<0.01***)
g2p FPR 10%	81.3	93.7	70.6	96.4	0.88 (p=0.40)	81.8	95	95.7	79.2	0.88 (p=0.30)	50	82.4	21.5	94.5	0.66 (p=0.15)
g2p FPR 15%	85	91.4	64.8	97	0.88 (p=0.48)	83.6	90	92	80	0.87 (p=0.34)	61.1	75.4	19.3	95.3	0.68 (p=0.20)
g2p FPR 20%	86.3	88.8	59	97.2	0.88 (p=0.41)	85.5	85	88.7	81	0.85 (p=0.23)	61.1	69.5	16.2	94.9	0.65 (p=0.13)
WebPSSM$_{SI/NSI}$-C[#]	82.5	89	58.3	96.5	0.86 (p=0.24)	85.5	77.5	83.9	79.5	0.82 (p=0.22)	61.1	81.3	23.9	95.6	0.71 (p=0.29)
11/25	45	98.1	81.5	90.5	0.72 (***p<0.01***)	47.3	97.5	96.3	57.4	0.72 (***p<0.01***)	11.1	98.4	40	92	0.55 (***p=0.01***)
11/24/25	47.5	96	68.9	90.7	0.72 (***p<0.01***)	52.7	95	93.5	59.4	0.74 (***p<0.01***)	22.2	97.3	44.2	92.9	0.60 (***p=0.048***)
11/25+V3 charge	57.5	97.7	82.3	92.5	0.78 (***p<0.01***)	52.7	95	93.5	59.4	0.74 (***p<0.01***)	22.2	98.4	57.2	92.9	0.60 (p=0.05)
HIVcoPRED (SAAC)	75	95.1	74.1	95.3	0.85 (p=0.19)	61.8	90	89.5	63.1	0.76 (p=0.20)	50	69	13.4	93.5	0.60 (***p=0.04***)
HIVcoPRED (SAAC+BLAST)	75	95.1	74.1	95.3	0.85 (p=0.19)	61.8	90	89.5	63.1	0.76 (p=0.20)	50	69	13.4	93.5	0.60 (***p=0.04***)
dsKernel	65	96.7	78.6	93.7	0.81 (***p=0.03***)	41.8	90	85.2	52.9	0.66 (p=0.08)	16.7	94.7	23.3	92.2	0.56 (***p=0.02***)
Lin *et al*	62.5	96.5	76.9	93.2	0.80 (***p<0.01***)	64.8	95.1	94.8	66.3	0.80 (p=0.07)	22.2	93	23.4	92.5	0.58 (***p=0.03***)

Sens (% sensitivity), Spec (% specificity), PPV (% positive predictive value) and NPV (% negative predictive value) were calculated as described in Supplementary Table 1 legend. P-values (two-tailed) of the difference in the area under the receiver operator characteristic curves (AUROC) between PhenoSeq and the indicated alternate genotypic algorithm are shown in parenthesis, calculated as described by Hanley *et al*[1]. P-values <0.05 were considered significant and are highlighted in bold italicized text. FPR, false positive rate. [#]The subtype C specific WebPSSM$_{SI/NSI}$ algorithm was used for subtype C HIV-1 predictions.

Supplementary Reference

1. Hanley JA & McNeil BJ (1982) The meaning and use of the area under a receiver operating characteristic (ROC) curve. *Radiology* 143(1):29-36.
